# Plasma extracellular vesicles carry immune system-related peptides that predict human longevity

**DOI:** 10.1007/s11357-024-01454-z

**Published:** 2024-12-18

**Authors:** Xin Zhang, Sisi Ma, Syeda Iffat Naz, Erik J. Soderblom, Constantin Aliferis, Virginia Byers Kraus

**Affiliations:** 1 Duke Molecular Physiology Institute, Duke University School of Medicine, Durham, NC 27701 USA; 2 Department of Orthopaedic Surgery, Duke University School of Medicine, Durham, NC 27701 USA; 3 Institute for Health Informatics, University of Minnesota, Minneapolis, MN USA; 4 Duke Proteomics and Metabolomics Core Facility, Duke University School of Medicine, Durham, NC USA; 5 Department of Medicine, Duke University School of Medicine, Durham, NC USA

**Keywords:** Extracellular vesicle, Longevity, Aging, Immune system, Muscle, Surface marker, Proteomics

## Abstract

Extracellular vesicles (EVs) play crucial roles in aging. In this National Institutes on Aging-funded study, we sought to identify circulating extracellular vesicle (EV) biomarkers indicative of longevity. The plasma EV proteome of 48 older adults (mean age 77.2 ± 1.7 years [range 72–80]; 50% female, 50% Black, 50% < 2-year survival, 50% ≥ 10-year survival) was analyzed by high-resolution mass spectrometry and flow cytometry. The ability of EV peptides to predict longevity was evaluated in discovery (*n* = 32) and validation (*n* = 16) datasets with areas under receiver operating characteristic curves (AUCs). Longevity-associated large EV (LEV) plasma subpopulations were mainly related to immune cells (HLA-ABC^+^, CD9^+^, and CD31^+^) and muscle cells (MCAD^+^ and RyR2^+^). Of 7960 identified plasma EV peptides (519 proteins), 46.4% were related to the immune system and 10.1% to muscle. Compared with short-lived older adults, 756 EV peptides (131 proteins) had a higher abundance, and 130 EV peptides (78 proteins) had a lower abundance in long-lived adults. Among longevity-associated peptides, 437 (58 proteins) were immune system related, and 12 (2 proteins) were muscle related. Using just three to five plasma EV peptides (mainly complement components C2-C6), we achieved high predictive accuracy for longevity (AUC range 0.91–1 in a hold-out validation dataset). Our findings suggest that immune cells produce longevity-associated plasma EVs and elucidate fundamental mechanisms regulating aging and longevity. EV longevity predictors suggest there may be merit in targeting complement pathways to extend lifespan, for instance, with any one of the multiple complement inhibitors currently available or in clinical development.

**Supplementary Information:**

The online version contains supplementary material available at 10.1007/s11357-024-01454-z.

## Introduction

To better understand successful aging and longevity, Miller et al. advocate the use of early warning signs to prevent a tipping point in stress resistance and resilience [1]. In clinical care, early warning signs are very often provided by blood tests used to monitor overall health or diagnose and monitor a medical condition. An abundant component of peripheral blood, extracellular vesicles (EVs), have been proposed as minimally invasive (no biopsy required) means of monitoring health, organ, and tissue specific processes because EVs carry surface markers and cargo from their parent cells that can reflect the health status, and disease states of the body [2–7]. In addition to their potential as markers of health and disease, EVs have recently been termed “very important particles” (VIPs) because of their crucial role in cellular communication [8]. Multiple subpopulations of circulating EVs, especially immune cell-associated EVs and their mitochondrial content, decline with aging [4, 9]. Heterochronic parabiosis of young and aged animals with active blood exchange suggests the ability of EVs from young animals to rejuvenate skeletal muscle and promote fracture healing in aged animals [2, 10], supporting a leading role of EVs in healthy aging and age-related diseases [11]. These findings support EVs as a novel class of biomarkers and tools for understanding the mechanisms underlying successful aging and longevity.

To develop early warning signs and understand factors influencing healthy aging in this National Institute on Aging (NIA) funded research, we sought to identify circulating EV biomarkers indicative of longevity to build a foundation for therapeutic strategies to extend healthspan and lifespan. We were especially interested in EVs of immune and muscle cell origin due to our recent observations in older adults of the NIA funded prospective Duke Established Populations for Epidemiologic Studies of the Elderly (D-EPESE) cohort [12], yielded from causal analyses, that lymphocytes, physical function and activity, and regular exercise were key potentiators of longevity, while neutrophils appeared to be attenuators of longevity, emphasizing the critical roles of immune and muscle functions in aging and longevity.

We hypothesized that plasma EVs related to immune and muscle systems carry functional cargo effectors that reflect and/or play a role in mediating longevity. To test our hypothesis, we used the patient-friendly biospecimen, plasma, for measurements of multiple EV characteristics – relative size distribution, 25 surface markers, and proteomic cargo– within a diverse subset of the D-EPESE cohort of older adults with 27 years of death data [12]. Based on extensive validation in our previous studies, the vesicles isolated from plasma using the same protocol as in this study met stringent criteria for identification as EVs. They comprised highly heterogeneous populations with a wide size distribution, bilayer structure, and typical EV morphology [2, 9]. These vesicles carried various surface markers, including traditional EV markers (CD9, CD81, and CD63) as well as cell and tissue-specific markers [9, 13, 14], along with cargo effectors such as cytokines [9, 13–15], mitochondria [9], microRNAs [16], and thousands of peptides [7, 14]. Furthermore, plasma EVs could promote the proliferation of recipient cells [14].

## Methods

### Study participants

For this study we selected 48 participants from the D-EPESE cohort [12], half long-lived (survived 10 or more years) and half short-lived (survived fewer than two years), gender (50% female, 50% male) and ethnicity balanced (50% White, 50% Black) (Table 1). One plasma specimen was analyzed for each participant. All samples and data were acquired with informed consent under IRB approval of Duke University.

**Table 1:** Demographic information and clinical measurements of 48 older adults from Duke-EPESE cohort

	Short-lived	Long-lived	*p* value
Demographic			
Sample number	24	24	
Age–mean (SD) (years)	77.42 (2.22)	77.04 (0.91)	0.4504
Age–range (years)	72–80	76–79	
Gender (female) %	50	50	
Race (Black) %	50	50	
BMI–mean (SD)	24.09 (4.18)	26.36 (4.68)	0.0826
Complete blood count–mean (SD)			
Red blood cell count (10^6^/mm^3^)	4.31 (0.74)	4.53 (0.66)	0.2904
Platelet count (10^3^/mm^3^)	213.67 (51.77)	243.96 (68.75)	0.0918
White blood cell count (10^3^/mm^3^)	6.20 (2.52)	6.22 (1.90)	0.9692
Neutrophils (10^3^/mm^3^)	4.24 (2.37)	3.82 (1.72)	0.4904
Lymphocytes (10^3^/mm^3^)	1.48 (0.51)	1.93 (0.59)	0.0068
Monocytes (10^3^/mm^3^)	0.31 (0.11)	0.34 (0.16)	0.4131
Eosinophils (10^3^/mm^3^)	0.14 (0.13)	0.11 (0.08)	0.3546
Basophils (10^3^/mm^3^)	0.04 (0.05)	0.03 (0.04)	0.3609
Neutrophils (%)	65.8 (9.5)	60.0 (9.0)	0.0367
Lymphocytes (%)	25.8 (8.4)	31.92 (7.8)	0.0111
Monocytes (%)	5.5 (2.5)	5.9 (2.3)	0.5517
Eosinophils (%)	2.5 (2.9)	1.8 (1.5)	0.3184
Basophils (%)	0.5 (0.5)	0.3 (0.5)	0.3867
Neutrophil–lymphocyte ratio (based on counts)	3.2 (2.5)	2.1 (1.0)	0.0410
Neutrophil–lymphocyte ratio (based on %)	3.2 (2.4)	2.1 (1.0)	0.0441
Physical function and activities–mean (SD)			
# Cognitive function related IADL items CAN do (telephone use, taking medications, managing finances)	2.4 (0.9)	2.9 (0.3)	0.0307
# Motor function related IADL items CAN do (traveling, shopping, preparing meals, doing housework)	2.4 (1.9)	3.8 (0.7)	0.0026
Rosow-Breslau 1: can do heavy housework (like washing windows, walls, or floors without help)	1.5 (0.5)	1.9 (0.3)	0.0046
Rosow-Breslau 2: able to walk up and down stairs (to the second floor without help, higher score = able to do)	1.6 (0.5)	1.9 (0.3)	0.0075
Rosow-Breslau 3: able to walk ½ mile without help (higher score = able to do)	1.5 (0.5)	2.0 (0.2)	0.0003

Standard *t*-test was performed to compare the difference between long-lived and short-lived participants; a *p* < 0.05 was considered as statistically significant. SD: standard deviation; mm^3^: cubic mm. *IADL* instrumental activities of daily living

### EV separation and characterization

**Fig. 1:**
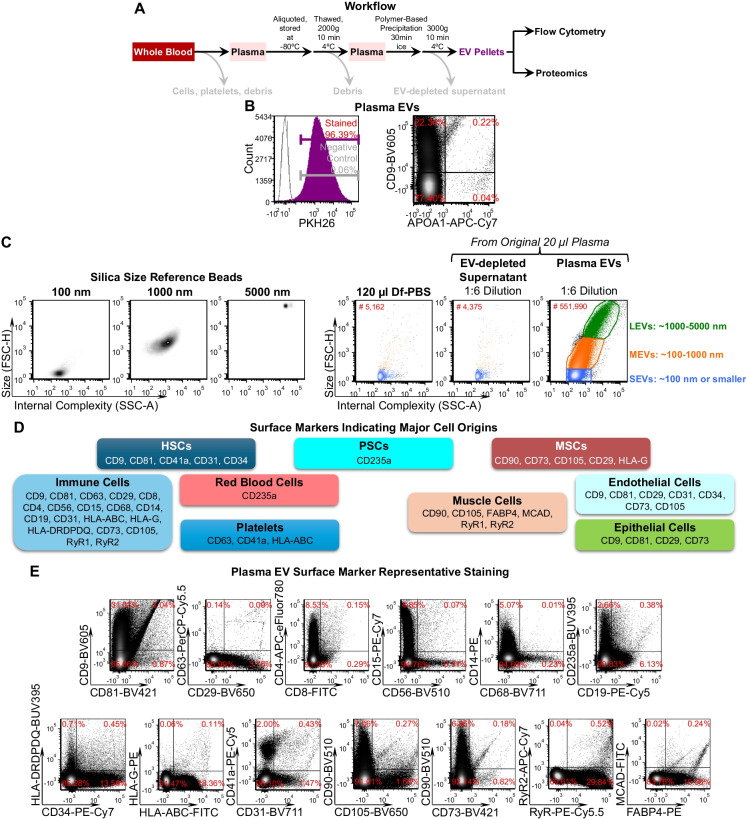
Plasma EV characterization. **A** The graph displays the workflow related to profiling EV for surface markers and proteomics. **B** The representative plot displays the percentage of PKH26^+^, CD9^+^, and APOA1^+^ particles in plasma EVs determined by high-resolution flow cytometry. **C** Silica size reference beads of mean size 100, 1000, and 5000 nm were used for size estimation; three major EV subsets were defined with following size ranges: large (LEVs), ~ 1000–5000 nm; medium (MEVs), ~ 100–1000 nm; and small (SEVs), ~ 100 nm or smaller; our reported EV sizes present an estimated approximal not actual sizes. **D** The graph displays the major cell origins of the tested surface markers. HSCs, hematopoietic stem cells; PSCs, pluripotent stem cells; MSCs, mesenchymal stem cells. **E** Representative dot plots present percentages of all tested surface markers in gated individual plasma EV subsets. RyR1^+^ population was defined as RyR^+^RyR2^−^, and RyR2^+^ population was defined as RyR^+^RyR2.^+^. FSC-H, forward scatter-height; SSC-A, side scatter-area. HLA-ABC, HLA-A, HLA-B, and HLA-C; HLA-DRDPDQ, HLA-DR, -DP and -DQ; RyR, ryanodine receptor; MCAD, M Cadherin, also known as cadherin 15 (CDH15) or muscle cadherin; FABP4, fatty acid binding protein 4

Following our previously reported protocols [9, 13, 15], fresh whole blood specimens collected in EDTA tubes were centrifuged at 3000 rpm for 15 min at 4°C to remove cells, platelets and debris; cell and platelet-depleted plasma was aliquoted and frozen at − 80°C. On the day of EV separation, frozen plasma was thawed and centrifuged at 2000 g for 10 min at 4°C to remove debris, then EVs were separated from plasma by polymer-based precipitation (ExoQuick, System Biosciences) following the manufacturer's instructions. EVs were characterized for size, lipid bilayer structure, and surface markers as we previously reported [7, 9, 13] (Fig. 1A). This methodology is preferred for isolating EVs of all sizes, enabling their subsequent characterization based on distinct size and surface markers.

### Mass spectrometry-based non-targeted proteomics

EVs isolated from 50 µl plasma were treated with 100 µl of 8 M urea, probe sonicated (yielding 802 ± 176 µg protein), then processed for proteomics by high-resolution mass spectrometry coupled with label-free quantification by the Duke Proteomics and Metabolomics Core Facility using a NanoAcquity UPLC system (Waters Corp) coupled to an Orbitrap Fusion Lumos high-resolution accurate mass tandem mass spectrometer equipped with a FAIMS Pro system (ThermoFisher Scientific) as previously described [7]. Peptide amino acid designations were assigned based on the SwissProt Human curated protein sequence database (downloaded Nov 2019). The relative quantitative expression value of each peptide (gravimetric (per ug) basis) was converted to a volumetric measure (per µl) by dividing the peptide value by the volume that yielded 20 µg EV-derived protein and multiplying by 2 to account for the twofold dilution of the plasma-derived EVs in 8 M urea.

STRING network analyses [17] were conducted to assess protein interactions and the functional enrichment of the EV proteome, focusing on aspects such as tissue expression patterns, Reactome pathways, and key biological processes. IPA core analysis [18] (QIAGEN) was conducted to identify key ingenuity Canonical Pathways, diseases and disorders, and master regulators associated with the longevity-linked proteome.

### Predictive modeling

Three predictive models of longevity were developed using immune system-related EV peptides identified through STRING network analyses [17]: (1) peptides without missing values (n = 1834) treated as continuous variables; (2) peptides with missing values (n = 1861) treated as binary variables; and (3) the combination of all peptides (n = 3695) treated as continuous variables imputing missing values as zero. We split the data into discovery (n = 32 participants, 16 long- and 16 short-lived) and holdout validation (n = 16 participants, 8 long- and 8 short-lived) datasets. We used the area under the receiver operating characteristic curve (AUC) to evaluate model performance. Further details are presented in Supplementary Methods.

### High resolution multicolor flow cytometry

Currently, scatter resolution alone –whether forward, side, or back scatter, using commercially available nanoscale or conventional flow cytometers–cannot fully distinguish EVs, particularly small EVs, from background noise. To effectively distinguish specific EV subsets, we combined EV size (forward scatter, FSC) and granularity (side and back scatter, SSC) parameters to characterize the relative size distribution of EVs for gating purposes. This was paired with fluorescence-conjugated antibodies to quantify the amounts and percentages of EVs carrying specific surface markers. A 50 µl plasma sample was utilized to profile each EV marker panel using flow cytometry as previously described [9, 13, 15]. We analyzed 3 EV marker panels, totaling 26 markers, designed utilizing FluoroFinder. EV pellets for each marker panel were resuspended in PBS (100 µl) double-filtered (df-PBS) using 100nm filters (EMD Millipore), and stained overnight at 4°C in the dark with shaking (300 rpm) with fluorescence-conjugated antibodies (1 µl each, 1:100 dilution) against human CD81, CD9, CD29, CD63, CD8, CD68, CD14, CD56, CD15, CD235a, CD41a, CD34, CD31, HLA-ABC, HLA-DRDPDQ, CD90, CD105, CD73 (BD Biosciences), CD4, CD19 and HLA-G (ThermoFisher Scientific), MCAD, FABP-4 (Santa Cruz Biotechnology), RyR, RyR2 and APOA1 (Novus Biologicals) in the presence of Brilliant Stain Buffer Plus (BD Biosciences). Using a method previously reported [14], separated EVs were resuspended in 100 µl staining buffer and stained using the PKH26 Red Fluorescent Cell Linker Midi Kit (1:200 dilution, MilliporeSigma) to label cell membranes. EVs were subsequently re-pelleted by polymer-based precipitation, and unbound dyes remaining in supernatants were removed. The high-resolution multicolor BD LSR Fortessa X-20 Flow Cytometer (BD Bioscience) was set up with the following key parameters: FSC voltage 410 and SSC voltage 210, SSC 200 as threshold to remove small debris particles, and low acquisition speed (~ 0.7 µl/second). Silica size reference beads of mean size 100 nm, 1000 nm (NanoXact Silica Nanospheres, VWR) and 5000 nm (Uniform Silica Microspheres, Bangs Laboratories) were used for size estimation. The fluorescence compensation, background, and positive signals were determined using unstained and single antibody/dye-stained df-PBS, EVs and UltraComp™ eBeads (ThermoFisher Scientific). The percentage (%) of plasma EVs carrying each surface marker were determined using flow cytometry; flow cytometric data analysis was performed using FCS Express 5 software (De Novo Software).

### Statistical analyses

The statistical analyses for this study included: (1) standard t-test for comparing complete blood count results between long-lived and short-lived participants; (2) Wilcoxon rank sum test to compare EV peptide quantitative expression values between the two groups (long-lived and short-lived participants), applying the Benjamini–Hochberg method to control the false discovery rate (FDR) at 0.01; (3) a Chi-squared test to compare EV peptide missing rates between the two groups, also controlling FDR at 0.01; (4) a Mann–Whitney U test to compare EV surface markers, quantified by flow cytometry, between the two groups; (5) Spearman correlations to analyze associations between EVs surface markers (percentage in LEVs, MEVs and SEVs), and the quantitative expression value of longevity-associated peptides. All tests were two-sided.

## Results

### Study participants

We selected 48 participants from the D-EPESE cohort (n = 1507 older adults) based on the following criteria: 24 individuals who died within 2 years (short-lived group), and 24 age-, sex- and ethnicity-matched individuals who survived ≥ 10 years (long-lived group) after the blood sample was obtained. This subsample of 48 older adults was balanced by age, gender and race, had mean age 77.2 ± 1.7 years (range 72–80), were 50% female, and 50% Black (Table 1). This subsample reflected the same baseline characteristics of long-lived versus short-lived older adults that we observed in the larger (n = 1507) Duke-EPESE cohort [12], including a higher number and percentage of lymphocytes, a lower percentage of neutrophils, a lower neutrophil to lymphocyte ratio (NLR), and better cognitive and physical function (by self-reported instrumental activities of daily living and Rosow-Breslau standardized questionnaire) (Table 1).

### EV characterization

Consistent with previous findings [2, 9], this study confirmed that most plasma EVs displayed a bilayer structure marked by PKH26^+^ signals (Fig. 1B), and low frequency of APOA1^+^ staining with a mean 0.9% APOA1^+^ particles across all 48 samples (representative staining in Fig. 1B). Notably, most APOA1^+^ particles co-expressed EV markers, such as CD9 (Fig. 1B), suggesting that these plasma particles may be partially derived from EVs. Using silica size-reference beads, we confirmed three major subsets of plasma EVs, consistent with our previous reports [9, 14], with the following estimated size ranges (Fig. 1C): large (LEVs), ~ 1000–5000 nm; medium (MEVs), ~ 100–1000 nm; and small (SEVs), ~ 100nm or smaller. Based on high-resolution multicolor flow cytometry, these plasma EVs carried typical EV markers and surface markers indicative of cell origins from several known stem cells and progenitor cells, hematopoietic cells including immune cells, activated pro-inflammatory fibroblasts, skeletal and cardiac muscle cells, epithelial cells, endothelial cells and adipocytes (Fig. 1D, E) [13, 15, 19–26].

### Comprehensive characterization of the plasma EV proteome in older adults

**Fig. 2:**
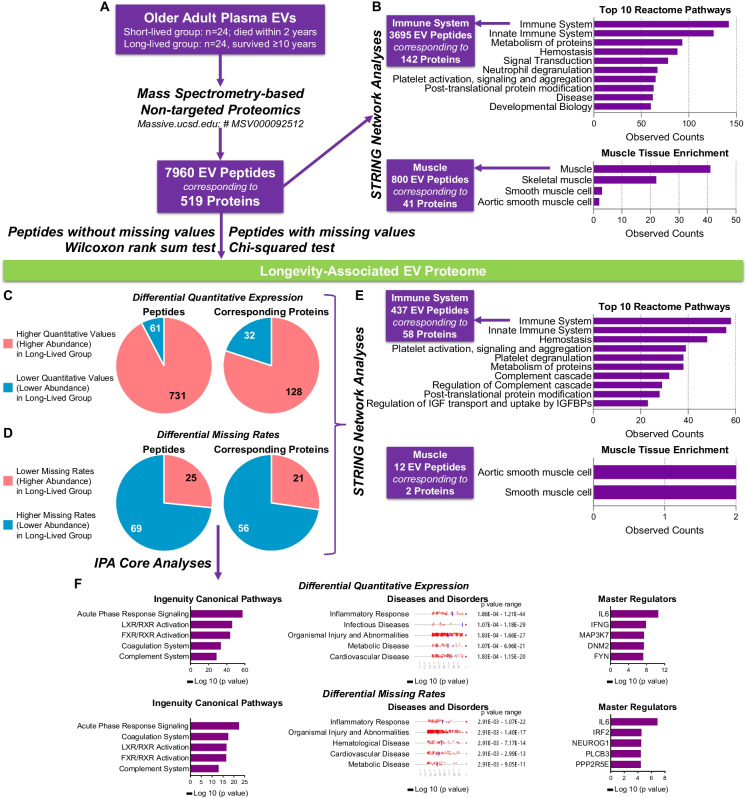
EV proteomic data yields longevity-associated peptides. Plasma EVs from 24 short-lived and 24 long-lived participants of D-EPESE cohort were profiled for proteomics by high-resolution mass spectrometry coupled with label-free quantitation. **A** This analysis on the older adult cohort plasma EVs identified 7960 peptides corresponding to 519 proteins. **B** STRING network analysis [17] identified immune system-related (3695 EV peptides corresponding to 142 proteins) and muscle-related (800 EV peptides corresponding to 41 proteins) proteome among the 7960 plasma EV peptides. C Peptides with missing rate > 0% in either group were excluded in quantitative expression analysis; Wilcoxon rank sum test was performed to compare the relative quantitative expression of each peptide between long-lived and short-lived participants; Benjamini–Hochberg procedure to control the false discovery rate at 0.01. The pie charts display 731 peptides (corresponding to 128 proteins) that have higher quantitative values (higher abundance), and 61 peptides (corresponding to 32 proteins) that have lower quantitative values (lower abundance) in long-lived than short-lived participants. **D** Chi-squared test was performed to compare the missing rate of each peptide between long-lived and short-lived participants; Benjamini–Hochberg procedure to control the false discovery rate at 0.01. The pie charts display 25 peptides (corresponding to 21 proteins) that have lower missing rate (higher abundance), and 69 peptides (corresponding to 56 proteins) that have higher missing rate (lower abundance) in long-lived than short-lived participants. **E** based on STRING network analysis [17], among the identified longevity-associated EV proteome, 437 EV peptides corresponding to 58 proteins were from immune system but only 12 EV peptides corresponding to 2 proteins were from muscle. **F** the graphs display IPA core analysis results of the top ingenuity canonical pathways, disease and disorders and master regulators of longevity-associated proteins identified based on their differential missing rates or differential quantitative expression between long-lived and short-lived participants

Mass spectrometry-based proteomic analysis with label-free quantification identified 7960 peptides corresponding to 519 proteins in plasma EVs from the D-EPESE subpopulation (n = 48) (Fig. 2A). STRING network analysis [17] identified 3695 immune system-related peptides, corresponding to 142 proteins, comprising 46.4% of all peptides, primarily from the innate immune system (Fig. 2B). Additionally, 800 peptides, corresponding to 41 proteins, mostly from skeletal muscle, accounted for 10.1% of all plasma EV peptides (Fig. 2B).

### Longevity-associated EV proteome in older adults

Longevity-associated EV peptides were categorized into two groups: those consistently quantified without missing values, and those with missing values in some participants due to low expression levels. Compared with short-lived older adults, long-lived older adults had a higher abundance of 731 peptides (128 proteins) without missing values, and 25 peptides (21 proteins) with missing values; while they had a lower abundance of 61 peptides (32 proteins) without missing values, and 69 peptides (56 proteins) with missing values (Fig. 2C-D, Supplementary Tables 1 and 2). In the longevity-associated proteome, STRING network analysis [17] identified 437 immune system-related peptides (58 proteins) and only 12 muscle-related peptides (2 proteins) (Fig. 2E).

Interestingly, based on Ingenuity Pathway Analysis (IPA) [18], the longevity-associated EV proteins for both groups of peptides (those without and with missing values) were involved in the same top canonical pathways, such as acute phase response signaling, coagulation system, and complement system, and the same top diseases and disorders, such as inflammatory response, cardiovascular disease, and metabolic disease. The top master regulator of these longevity-associated EV proteins was IL-6 (Fig. 2F).

We identified 44 longevity-associated EV proteins that were involved in the top canonical pathway—acute phase response signaling (Supplementary Fig. 1). Based on STRING network analysis [17], these proteins are highly interactive; 38 are enriched in extracellular exosome, a key type of SEVs, and 22 are enriched in immune system (Supplementary Fig. 1). Many of the longevity-associated EV proteins related to the acute phase response signaling pathway—such as FGA, FGB, FGG, FN1, and AMBP—are also associated with the severity of radiographic knee osteoarthritis [7].

### Immune system-related EV peptides were strong predictors of longevity

Given the established role of chronic inflammation as one of the twelve hallmarks of aging [27], and the dominance of immune system-related longevity predictors in the plasma EV proteome of older adults (Fig. 2), we developed models to predict longevity using the 3695 immune system-related EV peptides. The cohort was split into discovery (n = 32, 16 long- and 16 short-lived individuals) and holdout validation (n = 16, 8 long- and 8 short-lived individuals) datasets. Three predictive models were built: 1) using EV peptides without missing values (1834 peptides); 2) using EV peptides with missing values (1861 peptides) coded as binary variables (0 = missing, 1 = non-missing); and 3) using all EV peptides (3695 peptides) with missing values imputed as zero. All models, particularly the model without missing peptide values, demonstrated strong predictive accuracy with high cross-validation (CV) of the AUCs, and high hold-out-validation performances (CV AUCs range 0.996–0.997, hold-out validation AUCs range 0.91–1), indicating strong predictive power for longevity, independent of clinical variables.

**Fig. 3:**
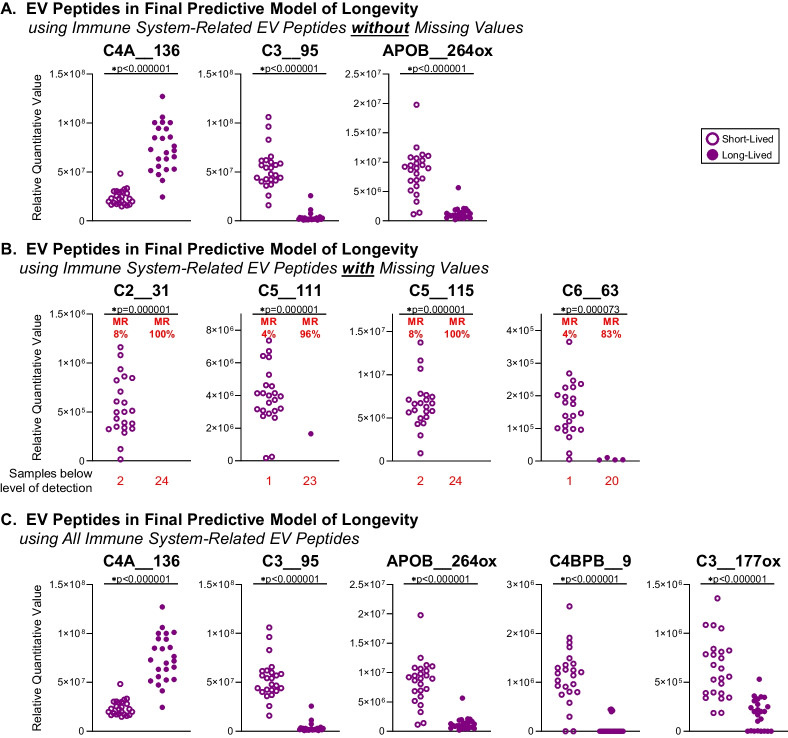
Immune system-related EV peptides were highly predictive of longevity. We built predictive models of longevity based on analyses of 3695 immune system-related EV peptides from the long- vs short-lived older adults. We split the data into discovery (*n* = 32 older adults, 16 long- and 16 short-lived) and holdout validation (*n* = 16 older adults, 8 long- and 8 short-lived) datasets. **A** The final model for the immune system-related EV peptides without missing (treated as quantitative) values yielded three EV peptides (C4A__136, C3__95, and APOB__264ox). Wilcoxon rank sum test was performed to compare the relative quantitative expression of each peptide between long-lived and short-lived participants; Benjamini–Hochberg procedure was used to control the false discovery rate at 0.01 (Supplementary Table 1). **B** The final model for the immune system-related EV peptides with missing (treated as binary 0 or 1) values yielded four highly predictive EV peptides (C2__31, C5__111, C5__115, and C6__63). Chi-squared test was performed to compare the missing rate of each peptide between long-lived and short-lived participants; Benjamini–Hochberg procedure to control the false discovery rate at 0.01 (Supplementary Table 2). MR, missing rate (percentage provided for short- and long-lived cohort). **C** The final model using the combined quantitative information for all 3695 immune system-related EV peptides, imputing missing values as zero, contained 5 peptides (C4a__136, C3__95, APOB__264ox, C4BPB__9, and C3__177ox). Wilcoxon rank sum test was performed to compare the relative quantitative expression of each peptide between long-lived and short-lived participants; Benjamini–Hochberg procedure was used to control the false discovery rate at 0.01

**Fig. 4:**
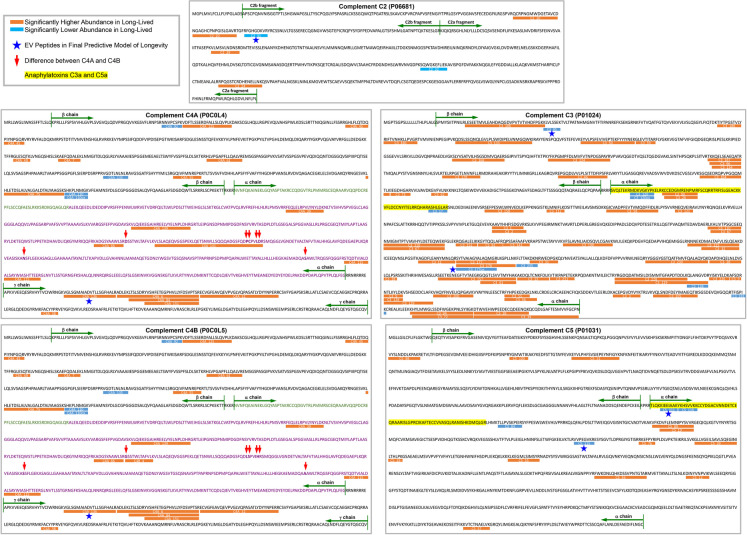
Mapping longevity-associated EV peptides in complement C2-5. Based on UniProt [28], the positions of longevity-associated EV peptides were mapped in the sequence of complement C2-5. Orange lines indicate the peptides with significantly higher abundance in long-lived older adults; blue lines indicate the peptides with significantly lower abundance in long-lived older adults; stars indicate the EV peptides in final predictive models of longevity, red arrows indicate the difference between C4 isotypes C4A and C4B; green arrows indicate starting and ending points of alpha (α), beta (β), and gamma (γ) chains; yellow highlighted sequences are anaphylatoxins C3a and C5a; in C4 (isotypes C4A and C4B) sequences, green and purple fonts indicate C4a and C4b fragments, respectively

The final model using EV peptides without missing values identified 3 peptide predictors (Fig. 3A), one positively correlated with longevity (complement C4 gamma [γ] chain [C4A__136]) and two negatively correlated with longevity (complement C3 beta [β] chain [C3__95] and apolipoprotein B [APOB__264ox] (Fig. 4). According to UniProt [28], the C4A__136 complement peptide is present in both complement C4 isotypes, C4A and C4B, which share 99% homology; they differ by only a few amino acids in the C4b fragment of the α chain, while their β and γ chains are identical (Fig. 4).

The final model using EV peptides with missing values identified 4 peptide predictors (Fig. 3B): complement C2b fragment (C2__31), complement C5 α chain (C5__111 and C5__115), and complement C6 (C6__63); all were negatively associated with longevity.

The final model using all 3695 EV peptides identified 5 peptides, the three peptides in the first model (Fig. 3A) plus an additional two peptides (Fig. 3C): complement C4 γ chain (C4A__136) – correlated positively with longevity, and complement C3 β chain (C3__95), apolipoprotein B (APOB__264ox), C4b-binding protein beta chain (C4BPB__9), and complement C3 α chain (C3__177ox) – correlated negatively with longevity.

Complement components in plasma EVs emerged as a cluster of biomarkers associated with longevity, exhibiting a complex pattern. Given the complex processing and diverse functions of complement components, it is unsurprising that EV peptide associations with longevity (both positive and negative) were tied to specific epitopes within these proteins, notably in C2–6, and particularly within the anaphylatoxins C3a and C5a (Fig. 3 and Fig. 4, Supplementary Tables 1 and 2). The more abundant EV peptides in long-lived compared with short-lived older adults were widely distributed across all chains of C4, C2, C3, and C5 (Fig. 4). In contrast, peptides with lower abundance in long-lived individuals were primarily localized to a specific (single) chain: all four low-abundant C4 peptides were in the C4 β chain, while all three low-abundant C5 peptides and seven out of eight low-abundant C3 peptides were in the α chains of C5 an C3 (Fig. 4). Notably, among these uniquely distributed low-abundance peptides in long-lived individuals, C3__120, C3__120ox, and C3__17 are components of the C3a anaphylatoxin, while C5__111 and C5__116 are components of the C5a anaphylatoxin (Fig. 4), both of which play major roles in mediating pro-inflammatory responses [29].

### Longevity-associated plasma EV subpopulations identified by their surface markers

**Fig. 5:**
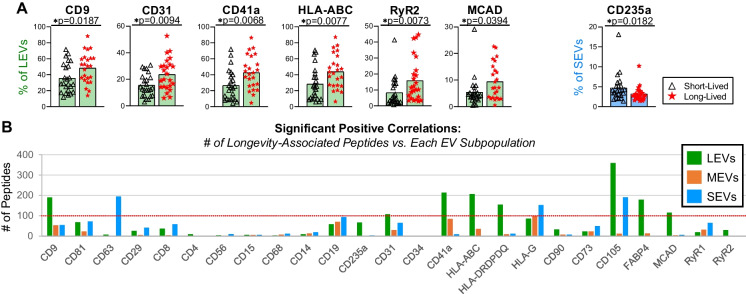
The frequency of multiple immune cell- and muscle-associated EV subpopulations was significantly higher in long-lived than short-lived participants. Plasma EVs from 24 short-lived and 24 long-lived participants of D-EPESE cohort were profiled with the indicated surface markers by high-resolution multicolor flow cytometry. **A** Mann–Whitney *U* test was performed to compare the difference between short- and long-lived subjects on the percentages of EVs carrying each tested surface marker in the gated LEVs, MEVs and SEVs; significant results defined by *p < 0.05. The graphs display the EV subpopulations with significantly different frequencies between long-lived and short-lived participants. **B** Spearman correlation was performed for assessing correlations between the percentage of EVs carrying each tested surface marker in the gated LEVs, MEVs, and SEVs and the quantitative expression value of each longevity-associated EV peptide that has differential quantitative value in long- and short-lived participants; significant results defined by *p < 0.05. The bar graphs display the number of longevity-associated EV peptide that were significantly positively correlated with the individual EV subpopulations

Compared with short-lived participants, long-lived participants had significantly higher percentages of LEVs from immune cells (HLA-ABC^+^, CD9^+^ and CD31^+^), muscle cells (MCAD^+^ and RyR2^+^) and hematopoietic stem cells (CD9^+^, CD31^+^ and CD41a^+^) (Fig. 1D) (Fig. 5A). In contrast, they exhibited a lower percentage of SEVs from red blood cells and pluripotent stem cells (Fig. 1D), as indicated by the CD235a^+^ subpopulation (Fig. 5A).

The frequencies of immune-related plasma LEV and SEV subpopulations were significantly positively correlated with over 100 longevity-associated EV peptides including the following: LEVs carrying CD9, CD31, CD41a, HLA-ABC, MCAD, HLA-DRDPDQ, CD105 and FABP4; and SEVs carrying CD63, CD105 and HLA-G (Fig. 5B). Specific longevity-associated LEV subpopulations (CD9^+^, CD31^+^, CD41a^+^, HLA-ABC^+^, MCAD^+^ and RyR2^+^ LEVs, Fig. 5A) were positively correlated with higher-abundance peptides, and negatively correlated with lower-abundance peptides in long-lived compared to short-lived older adults (Supplementary Table 3). This suggests that cells expressing the same surface markers, particularly immune cells, contribute to the longevity-associated plasma EV subpopulations and their peptide cargo.

## Discussion

An aged, senescent immune system drives systemic aging and development of many age-related diseases and leads to increased morbidity and mortality [30, 31]. Among our 7960 detected plasma EV peptides, 3695 EV peptides were related to the immune system, primarily innate immunity. The longevity-associated EV peptides were primarily involved in inflammatory response, cardiovascular disease, and metabolic disease. Three predictive models yielded high AUCs (0.91–1) with remarkably few (range 3–5) EV peptides required for robust longevity predictions.

A large proportion of the proteomic predictors of longevity were derived from the complement system that has demonstrated roles in aging, longevity and age-related diseases [32]. We also identified high expression of the complement system proteome in osteoarthritic synovial fluid EVs (8.2% of all synovial fluid EV peptides) and their association with knee osteoarthritis severity [7], supporting the involvement of the complement system in the pathogenesis of age-related diseases [33, 34]. This study identified multiple complement system-related peptides in plasma EVs that were linked to, or predictive of, longevity status in older adults.

We hypothesize that therapeutic benefits of complement inhibitors might be inferred from the distribution of the identified longevity-associated EV peptides within complement sequences and their link to longevity. To explore this, we analyzed specific epitopes in key complement components. Our findings suggest that higher abundance of EV peptides across all chains of complement C2–5 in long-lived versus short-lived older adults, may enhance defense against infections [32]. In contrast, only a limited number with reduced abundance were located in the C4 β chain and α chains of C3 and C5, particularly within the pro-inflammatory anaphylatoxins C3a and C5a, which are critical in inflammation-associated diseases and conditions [29]. Selectively inhibiting or removing these peptides, particularly in C3a and C5a, might mimic the phenotype observed in long-lived older adults and promote lifespan extension.

The C4 protein, a trimer of α, β, and γ chains [35, 36], activates early in the complement cascade [32]. While the roles of C3a and C5a in inflammation are well-defined, C4a's role remains unclear, with none of the longevity-associated peptides derived from C4a. We observed that six peptides in the C4 α chain, localized in the C4b fragment (a component that mediates clearance of exogenous pathogens [37]), and six in the C4 γ chain, were consistently more abundant in long-lived individuals. Conversely, peptides with reduced abundance in long-lived adults were found only in the C4 β chain. Interestingly, consistent with these findings, higher levels of a peptide from the beta chain of Complement Component 4 Binding Protein Beta (C4BPB), which inhibits the function of C4b and thereby prevents complement-mediated clearance of exogenous pathogens [38], was associated with reduced longevity. These findings suggest that higher levels of C4b and C4 γ chain peptides in plasma EVs may promote or mark longevity, while targeting specific peptides in the C4 β chain could offer anti-aging benefits. To date, no C4 inhibitors are in development, highlighting an opportunity for novel C4-targeting therapies.

Several complement inhibitors are in clinical development for age- and immune system-related diseases [39–41] including: Zimura, a C5 inhibitor for age related macular degeneration; Eculizumab, another C5 inhibitor for lupus nephritis and glomerulonephritis; IFX-1 (Vilobelimab), a C5a inhibitor for septic shock and inflammation; Compstatin, a C3 inhibitor for sepsis and systemic inflammatory response syndrome, and age-related macular degeneration; and Mirococept, a C3 convertase inhibitor for rheumatoid arthritis, inhibiting C3 cleavage to C3a and C3b. Based on our findings, IFX-1 and Mirococept may have longevity-promoting effects by inhibiting C5a and C3a, respectively. Further study, particularly with available complement-targeting drugs, is needed to understand longevity-related pathways and potential long-term effects.

In this study, long-lived older adults had significantly higher baseline percentages of multiple plasma LEV subpopulations associated with immune cells and muscle cells. This conclusion was also borne out by the EV proteome data identifying immune cells as major contributors to longevity-associated plasma EVs. To date, most studies in the EV literature have focused on SEVs, while LEVs remain under-studied, resulting in limited information about LEVs. LEVs and SEVs have been shown to possess both shared and unique peptides [42, 43]. A previous study showed that LEVs (1–10 μm in diameter) contain proteins and small RNAs [44], consistent with our earlier findings that EVs derived from skeletal muscle tissue culture supernatant (approximately 100–6000 nm in diameter) carry hundreds of microRNAs [16]. LEVs originating from natural killer cells can modulate surface marker expression and function of THP-1 cells (a monocyte/macrophage cell line)[45], while some LEVs can even encapsulate, transport and release SEVs [46]. Although the extent of this phenomenon across different cell types remains unclear, it suggests that these particles may play a signaling role rather than serving solely as a means of removing cellular waste. This underscores the importance of studying EVs across all size ranges, as demonstrated in this study.

There are certain limitations associated with this study. The EV longevity predictor results need to be treated with caution, since the sample size for discovery was relatively small, and need further validation in larger samples and ideally in cohorts that include individuals younger than age 71 years. There is still no “perfect” high-yield, high-purity method for isolating EVs. All EV isolation methods result, to different degrees, in co-isolation of non-EV particles [47]. Many methods focus on small EVs only [47]. Since there is not enough evidences to exclude medium and large EVs from studies of longevity, we favored a holistic approach that relied on polymer-based precipitation to ensure high yields of diverse EVs of all sizes from small volumes of human specimens with stringent characterization to validate their identity as EVs, including dynamic light scattering, high-resolution multicolor flow cytometry, nanoparticle tracking analysis, and transmission electron microscopy [2, 9, 15]. Although storage time is a potential limitation of this study, sample processing for proteomics requires trypsin digestion of proteins. For this reason, this analytical methodology can tolerate a certain level of protein degradation. Our prior non-targeted proteomic analysis of plasma EVs of healthy donors revealed no significant changes in peptide concentrations with one, three or six serial freeze–thaw cycles compared with baseline [14]. These results are consistent with previous studies demonstrating stability of EV miRNAs from samples stored for 5–12 years [48, 49]. Compared to fresh plasma-derived SEVs, frozen plasma-derived SEVs showed altered morphology but maintained their biologic activity [50]. Similarly, our prior study demonstrated that EVs from frozen plasma significantly promoted the proliferation of recipient cells [14]. While EVs may lose some biomarkers during long-term storage, the plasma samples in our study were all collected in 1992 [12] and stored at − 80°C, with EV isolation performed simultaneously across all samples. This standardized approach minimized the likelihood that storage conditions contributed to differences in EV protein identification.

## Conclusions

In plasma EVs from older adults, 46.4% of 7960 identified peptides (3,695 peptides from 142 proteins) were immune system-related, predominately from the innate immune system, while 10.1% (800 peptides from 41 proteins) were muscle-related, primarily skeletal muscle. Among 880 longevity-associated EV peptides, 437 (from 142 proteins) were immune-related, and 12 (from 2 proteins) were muscle-related. Remarkably, using just three to five plasma EV peptides–mainly complement components C2-C6–achieved high predictive accuracy for longevity. These findings suggest that EVs derived from immune cells contribute significantly to the pool of longevity-associated plasma EVs. This work provides a basis for further investigating the mechanisms of these longevity-associated plasma EV biomarkers in longevity and developing therapeutic targets to promote healthy aging and longevity.

## Supplementary Information

Below is the link to the electronic supplementary material.

Supplementary file1 (DOCX 25 KB)

Supplementary file2 (DOCX 1904 KB)

Supplementary file3 (XLSX 129 KB)

Supplementary file4 (XLSX 20 KB)

Supplementary file5 (XLSX 53 KB)

## Data Availability

All data needed to evaluate the conclusions in the paper are present in the paper and/or the Supplementary Materials. All proteomic data for the present study is available at massive.ucsd.edu (MSV000092512).
