# Supplementary material for: Plasma extracellular vesicles carry immune system-related peptides that predict human longevity
Source: GeroScience. 2024 Dec 18;47(2):1455–69. doi: 10.1007/s11357-024-01454-z (PMC11979029; doi:10.1007/s11357-024-01454-z)
Supplement: Supplementary file 1 — Supplementary file1 (DOCX 25 KB) [file 11357_2024_1454_MOESM1_ESM.docx]

**Supplementary Methods**

**Predictive Modeling**

We built predictive models of 2-year vs. 10-year longevity based on analyses of 3695 immune system-related EV peptides (1834 without any missing value, and 1861 with some missing values) from the long- vs short-lived older adults. We split the data into discovery (n=32 participants, 16 long- and 16 short-lived) and holdout validation (n=16 participants, 8 long- and 8 short-lived) datasets. We analyzed the EV peptides without missing values separately from EV peptides with missing values. We also analyzed the combined information in the peptides with and without missing values. We built three sets of models using the following features: (1) the quantitative expression of peptides without any missing values; (2) the missingness pattern of the peptides with missing values, where non-detected (missing) values were coded as 0 and detected (non-missing) values coded as 1; (3) features representing information from the missing and non-missing peptides, the expression value all 3695 peptides with missing values imputed as zeros (imputing with zero reflects the assumption that missingness is due to expression below the level of quantification by mass spectrometry). We used AUCs to evaluate the model performance. We applied the repeated nested five-fold cross-validation (NCV) protocol on the discovery dataset. The inner loop of the NCV conducts model selection whereas the outer loop of the NCV performs performance evaluation. The NCV protocol selects the best performing model while avoiding overfitting and produces unbiased performance estimates. Repeating the analysis reduces the variance of the estimator. The NCV examines the combinations of classification methods and feature selection methods for the best model[1]. The sets of classification methods examined were logistic regression, random forest, boosted trees, and support vector machine. The latter three methods employ various forms of regularization and capacity control to avoid overfitting. To further reduce model complexity, we used GLL feature selection, a Markov Boundary induction method, which has strong theoretical properties for parsimony and optimal predictivity. Moreover, extensive benchmarking studies on biomedical data demonstrate the strong empirical performance of GLL, that produces the most compact and predictive maximum feature sets across large numbers of tested datasets, including data with small sample sizes[2, 3].

To compare the performance of the three different models, we applied a label permutation test to the discovery data cross validation[4] and Delong’s test on the hold-out validation results[5]. The label permutation test[6] statistically examines: (a) if the overall analysis and modeling protocol was biased and; (b) tests the performance on the original dataset against the null, where features do not contain information for predicting the target. Specifically, we randomly permuted the order of the outcome (long- or short- lived) to remove the relationship between the outcome and the peptide features. We then applied the entire modeling procedure to the permuted dataset to obtain the predictive performance. One hundred permuted datasets were generated resulting in one hundred predictive performances, which forms the empirical null distribution. The resulting empirical distribution means were 0.487, 0.485, and 0.503 for the three models respectively (close to AUC=0.5, indicating no signal), demonstrating that our modeling procedure was unbiased. Comparing the predicted performance on the original data to the distribution for the random model establishes the statistical significance.

**References**

1. Duda RO, Hart PE, Stork DG: Pattern Classification. Wiley; 2012.

2. Aliferis CF, Statnikov A, Tsamardinos I, Mani S, Koutsoukos XD: Local Causal and Markov Blanket Induction for Causal Discovery and Feature Selection for Classification Part I: Algorithms and Empirical Evaluation. Journal of Machine Learning Research 2010, 11:171-234.

3. Aliferis CF, Statnikov A, Tsamardinos I, Mani S, Koutsoukos XD: Local Causal and Markov Blanket Induction for Causal Discovery and Feature Selection for Classification Part II: Analysis and Extensions. Journal of Machine Learning Research 2010, 11:235-284.

4. Bandos AI, Rockette HE, Gur D: A permutation test sensitive to differences in areas for comparing ROC curves from a paired design. Statistics in Medicine 2005, 24:2873-2893.

5. Delong ER, Delong DM, Clarkepearson DI: Comparing the Areas under 2 or More Correlated Receiver Operating Characteristic Curves - a Nonparametric Approach. Biometrics 1988, 44:837-845.

6. Ojala M, Garriga GC: Permutation Tests for Studying Classifier Performance. Journal of Machine Learning Research 2010, 11:1833-1863.
