# Supplementary material for: Plasma extracellular vesicles carry immune system-related peptides that predict human longevity
Source: GeroScience. 2024 Dec 18;47(2):1455–69. doi: 10.1007/s11357-024-01454-z (PMC11979029; doi:10.1007/s11357-024-01454-z)
Supplement: Supplementary file 2 — Supplementary file2 (DOCX 1904 KB) [file 11357_2024_1454_MOESM2_ESM.docx]

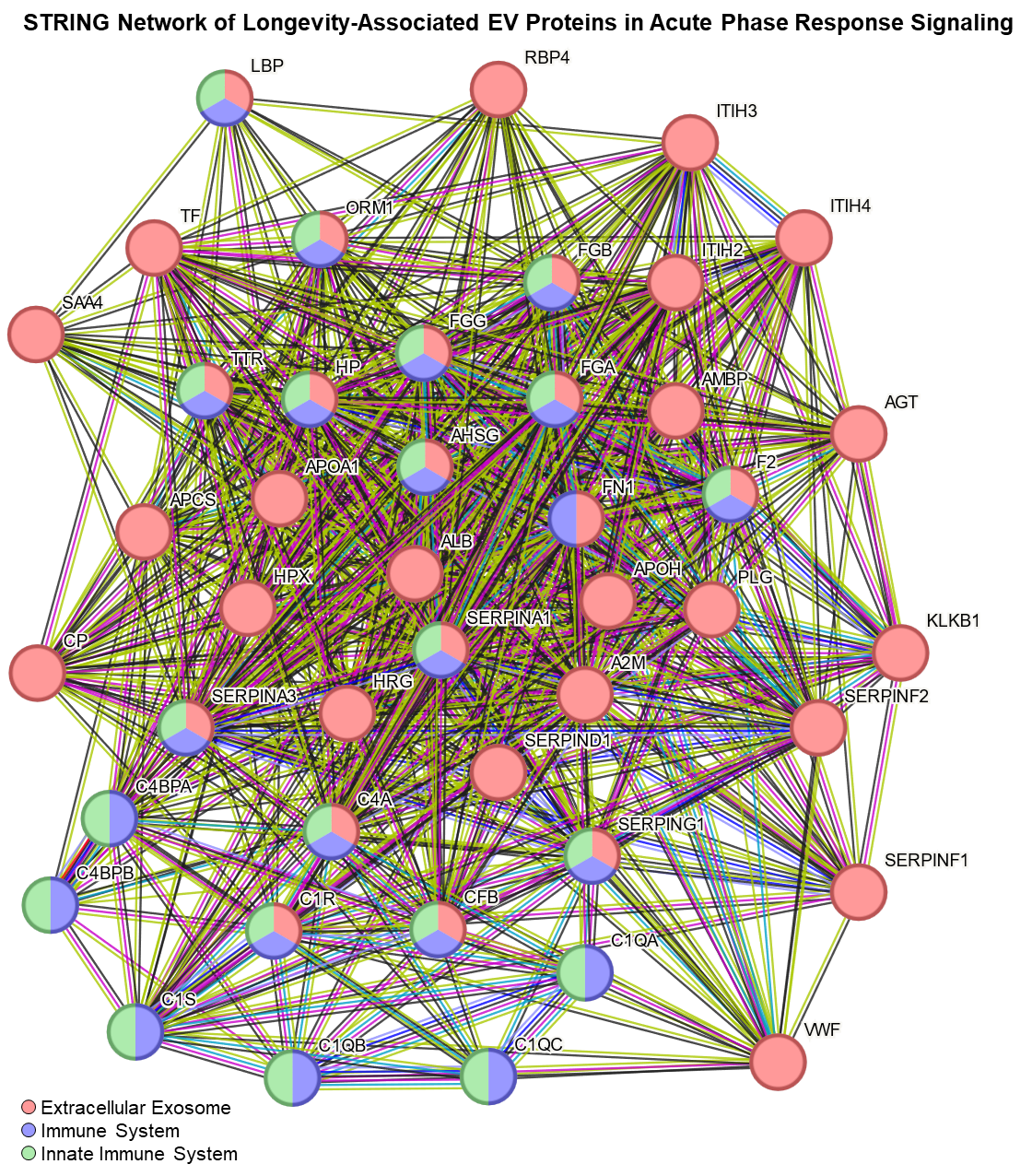


**Supplementary Figure 1.** Longevity-associated EV proteins were involved in the top canonical pathway—acute phase response signaling. The STRING Network displays the interaction of the 44 identified longevity-associated EV proteins in the acute phase response signaling pathway. The functional enrichment indicated by the colors of each node in the network are detailed in the legend. The edges illustrate protein associations, both functional and physical, all supported by evidence. The reported pathways exhibited a False Discovery Rate <0.05.
